# A retrospective study on the efficacy of the ERAS protocol in patients who underwent laparoscopic left and right colectomy surgeries

**DOI:** 10.3389/fsurg.2024.1395271

**Published:** 2024-06-25

**Authors:** Xuemei Zhao, Siyu Jin, Mingxiu Peng, Jingjing Wang

**Affiliations:** ^1^ Outpatient Department, Chengdu Shang Jin Nan Fu Hospital/Shang Jin Hospital of West China Hospital, Sichuan University, Chengdu, Sichuan, China; ^2^ Department of Neurology, West China Hospital, Sichuan University, Chengdu, Sichuan, China

**Keywords:** colorectal cancer, Enhanced Recovery After Surgery, postoperative rehabilitation, postoperative complications, surgery

## Abstract

**Objective:**

Retrospective analysis and comparison of the effects of Enhanced Recovery After Surgery (ERAS) protocol for patients having left and right colectomy surgeries.

**Method:**

Out of the patients admitted to Chengdu Shang Jin Nan Fu Hospital and West China Hospital from December 2019 to December 2022, a total of 498 who met the inclusion criteria were selected, 255 with right colectomy(RC) and 243 with left colectomy (LC). Under the conditions of strict compliance with ERAS protocol, the relevant physical indexes of RC and LC, including postoperative rehabilitation (especially median post-operative stay) and complications (especially prolonged postoperative ileus, PPOI), were statistically analyzed and compared.

**Results:**

In terms of intraoperative variables, fluid doses were higher in the LC group than in the RC group (*P* < 0.05), and there was no significant difference between them in terms of operative time, blood loss, need for open surgery, peritoneal contamination, epidural catheter placement, or opioid use (*P* > 0.05). Compared with the RC group, the LC group had a higher intake of oral liquid at the second postoperative day (POD), and faster first flatulence (*P* < 0.05). 30 (11.76%) RC patients required nasogastric tube insertion, while only 3 (1.23%) patients in the LC group required the same (*P* < 0.05). Prolonged postoperative ileus (PPOI) occurred in 48 (18.82%) and 29 (11.93%) patients in the RC and LC groups, respectively (*P* < 0.05). No significant differences in terms of postoperative complications or length of hospital stay (LoS). stay were observed.

**Conclusion:**

As the location of colon cancer changes, the effectiveness of ERAS also varies. More personalized and precise ERAS protocols can reduce the incidence of postoperative complications and promote rapid recovery after surgery.

## 1 Introduction

Colorectal cancer (CRC) is the most common tumor of the digestive system, and estimates of new cases and deaths from CRC in the United States in 2024 are 152,810 and 53,010, respectively (1). At present, the main treatment for CRC is surgical tumor removal, and there are mainly two ways to do that depending on the location of the tumor, namely left colectomy (LC) and right colectomy (RC) (2, 3). Depending on tumor characteristics and surgeon experience, there are still debates about what type of surgery to choose to treat transverse colon cancer (4). Enhanced Recovery After Surgery (ERAS) is widely used due to its applicability and safety. Many studies have shown that ERAS can help improve prognostic recovery after colorectal surgery. This multimodal stress minimization method has been repeatedly proven to be able to reduce morbidity after CRC surgery, improve postoperative recovery, and shorten median median post-operative stay (5, 6). ERAS aims to bring patients back to their preoperative state quickly after surgery through multiple efforts such as minimizing perioperative fasting, encouraging exercise, and strict pain control (7). However, it is worth exploring whether we should implement different ERAS protocols depending on the location of the colon tumor. The goal of this study is to initially explore whether there are differences in the ERAS process of patients undergoing LC or RC colon cancer surgeries.

## 2 Materials and Methods

### 2.1 Object of study

A multi-center retrospective study based on anonymized data collected. According to the The Societies present the Reporting on ERAS Compliance, Outcomes, and Elements Research (RECOvER) Checklist, our center started to systematically implement the standardized ERAS protocol for elective colectomy from 2019. Since then, we have collected demographic and perioperative data as well as functional and clinical results through bedside patient diaries and electronic charts and paid follow-up visits postoperatively (for at least 30 days) to all patients.

**Figure 1:**
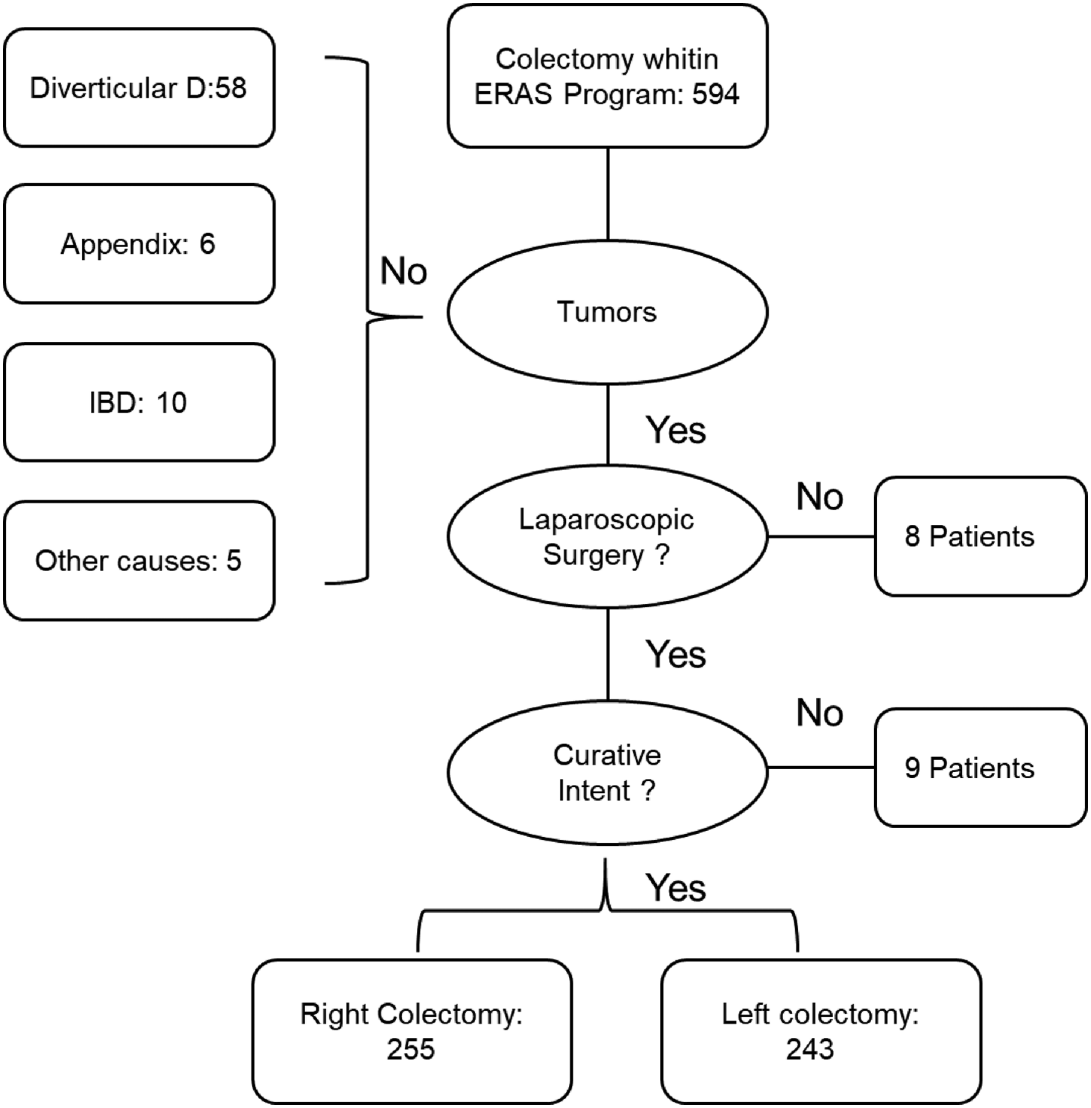
Flowchart to select the study population.

Patients admitted to Chengdu Shang Jin Nan Fu Hospital and West China Hospital from December 2019 to December 2022 who underwent elective colectomy (mainly laparoscopic RC or LC for cancer treatment) were selected and treated according to the standardized ERAS protocol, shown in Figure 1. Prolonged postoperative ileus (PPOI) was the primary endpoint, and the secondary endpoints was median post-operative stay (8, 9). This study involving human participants were reviewed and approved by the ethics committee of Chengdu Shang Jin Nan Fu Hospital (EC20200029), and were conducted according to established ethical guidelines.

Exclusion criteria: (1) Tumor emergencies such as intestinal obstruction, perforation, and bleeding; (2) Multiple cancers, neoadjuvant chemoradiotherapy, extensive tumor metastasis found during surgery, etc.; (3) Inability to tolerate surgery due to severe cardiopulmonary insufficiency.

### 2.2 Method

#### 2.2.1 Surgical intervention

According to oncological principles, all laparoscopic surgeries are performed in the presence of at least one senior colorectal surgeon, with a 7–10 cm excisional margin on both sides of the tumor, and central ligation and lymph node resection along the corresponding blood vessels. Tumors that occur in the right transverse colon or proximal transverse colon were treated with RC (resection of the terminal ileum, right colon, and partial transverse colon, followed by ileostomy). Left-sided colon tumors were treated with LC (sigmoidectomy or left-sided colectomy, followed by first-stage colorectal anastomosis). All LCs were end-to-end colorectal anastomoses performed with conventional double staplers (10, 11).

ERAS protocol was summarized based on relevant literature and previous experience, as shown in Table 1.

**Table 1:** ERAS protocols (developed based on literature review and previous experience) (12–14).

ERAS item	Content
Preadmission information, education and counselling	Timely individualized education, including surgical precautions. Proper and complete information may reduce anaesthesia- and surgery-related anxiety and subsequent pain.
Preoperative optimisation	Risk assessment, smoking cessation and avoiding alcohol abuse.
Prehabilitation	Prehabilitation shows promising results in recovery of functional capacity and may reduce complications after colorectal surgery
Preoperative nutritional care	Preoperative routine nutritional assessment offers the opportunity to correct malnutrition and should be offered. Preoperatively, patients at risk of malnutrition should receive nutritional treatment preferably using the oral route for a period of at least 7–10 days.
Prevention of nausea and vomiting	A multimodal approach to PONV prophylaxis should be considered in all patients. Patients with 1–2 risk factors should ideally receive a two-drug combination prophylaxis using first-line antiemetics. Patients with ≥2 risk factors undergoing colorectal surgery should receive 2–3 antiemetics.
Pre-anaesthetic medication	Pharmacologic anxiolysis with long- or short-acting sedative medication (especially benzodiazepines and especially in the elderly) should be avoided if possible before surgery.
Antimicrobial prophylaxis and skin preparation	Intravenous antibiotic prophylaxis should be given within 60 min before incision as a single-dose administration to all patients undergoing colorectal surgery. In addition, in patients receiving oral mechanical bowel preparation, oral antibiotics should be given. No recommendation for the use of oral antibiotic decontamination can be given for patients having no bowel preparation.
Bowel preparation	Oral administration of metronidazole and levofloxacin 2 days before surgery, liquid intake 1 day before surgery, and oral administration of colonic cleansing powder after surgery.
Preoperative fluid and electrolyte therapy	Patients should reach the anaesthetic room in as close a state to euvolaemia as possible and any preoperative fluid and electrolyte excesses or deficits should be corrected.
Preoperative fasting and carbohydrate loading	Patients undergoing elective colorectal surgery should be allowed to eat up until 6 h and take clear fluids including CHO-maltodextrin, up until 2 h before initiation of anaesthesia.
Standard anaesthetic protocol	Short-acting anaesthetics, cerebral monitoring are used to improve recovery and reduce the risk for postoperative delirium, monitoring of the level and complete reversal of neuromuscular block is recommended.
Intraoperative fluid and electrolyte therapy	The goal of perioperative fluid therapy is to maintain fluid homoeostasis avoiding fluid excess and organ hypoperfusion. A perioperative near-zero fluid balance approach should be preferred.
Preventing intraoperative hypothermia	Reliable temperature monitoring should be undertaken in all colorectal surgical patients and methods to actively warm patients to avoid inadvertent perioperative hypothermia (IPH) should be employed.
Surgical access	A minimally invasive approach to colon and rectal cancer has clear advantages for improved and more rapid recovery, reduced general complications, reduced wound-related complications including incisional hernia and fewer adhesions.
Drainage of the peritoneal cavity and pelvis	Pelvic and peritoneal drains show no effect on clinical outcome and should not be used routinely.
Nasogastric intubation	Postoperative nasogastric tubes should not be used routinely; if inserted during surgery, they should be removed before reversal of anaesthesia.
Postoperative analgesia	Avoid opioids and apply multimodal analgesia in combination with spinal/epidural analgesia or TAP blocks when indicated.
Thromboprophylaxis	Patients undergoing major colorectal surgery should have (I) mechanical thromboprophylaxis by well-fitting compression stockings and/or intermittent pneumatic compression until discharge and (II) receive pharmacological prophylaxis with LMWH once daily for 28 days after surgery.
Urinary drainage	Routine transurethral catheterisation is recommended for 1–3 days after colorectal surgery. The duration should be individualised based on known risk factors for retention: male gender, epidural analgesia and pelvic surgery.
Prevention of postoperative ileus	A multimodal approach to minimise the development of postoperative ileus include: limit opioid administration through use of multimodal anaesthesia and analgesia techniques, use minimally invasive surgical techniques (when feasible), eliminate routine placement of nasogastric tubes and use goal-directed fluid therapy.
Postoperative glycaemic control	Several interventions in the ERAS protocol prevent insulin resistance, thereby improving glycaemic control with no risk of causing hypoglycaemia. For in patients, insulin should be used judiciously to maintain blood glucose as low as feasible with the available resources.
Postoperative nutritional care	Most patients can and should be offered food and oral nutritional supplement (ONS) from the day of surgery. Perioperative immunonutrition in malnourished patients is beneficial in colorectal cancer surgery.
Early mobilisation	Early mobilisation through patient education and encouragement is an important component of enhanced recovery after surgery programmes.

#### 2.2.2 Data source

The dataset used consists of multiple items which are summarized, including but not limited to time to first flatulence, stool, activity, postoperative visual analogue scale (VAS), nausea and vomiting, time to tolerance with solid food, complications, opioids (as needed), and median post-operative stay. The prerequisites for ERAS patients to be discharged include: (1) no evidence of complications; (2) tolerance to soft diet (SD); (3) unassisted walking; (4) good pain control with oral administration of drugs. RECOvER Checklist was shown in Table 2.

**Table 2:** RECOvER checklist.

Item		Recommendation pag
Title	Title	Application of ERAS protocol in left and right colectomy surgeries.
Introduction	Background	The effects of ERAS protocols on colon tumor recovery in different location of colon are uncertain.
	Guidelines	Pędziwiatr, et al. (12)
	Outcomes	PPOI was the primary endpoint, and the secondary endpoints was median post-operative stay, postoperative complication.
Methods	IRB approval	The ethics committee of West China Hospital # EC20200029.
	Study design	Retrospective cohort study.
	Setting	Single institution, large general hospital with stable group of surgeons during the study Period.
	Timing	Patients included from December 2019 to December 2022, events assessed daily from surgery to discharge, all patients followed until 4-week postoperative visit.
	Participants	Inclusion criteria: 18+ years old with colon tumor, participating in the enhanced recovery protocol, undergoing laparoscopic radical surgery, not admitted to ICU postoperatively. Exclusion criteria: (1) Tumor emergencies such as intestinal obstruction, perforation, and bleeding; (2) Multiple cancers, neoadjuvant chemoradiotherapy, extensive tumor metastasis found during surgery, etc.; (3) Inability to tolerate surgery due to severe cardiopulmonary insufficiency.
	Enhanced recovery protocol	Details are shown in Table 1.
	Enhanced recovery auditing	All enhanced recovery elements charted by physician assistant into Enhanced Recovery Interactive Audit System (EIAS)
Results	Patient population	See Table 3.
	Intraoperative results	See Table 4.
	Postoperative functional recovery	See Table 5.
	Postoperative complication	See Figure 2.
Discussion	Context	As the location of colon cancer changes, the effectiveness of ERAS also varies. More personalized and precise ERAS protocols can reduce the incidence of postoperative complications and promote rapid recovery after surgery.
	Limitations	First, as a small-sample, single-center retrospective study, this research covers only a small number of cases, and the conclusion needs to be validated by further multi-cohort studies. Second, in terms of observation indicators, this study lacks comprehensiveness due to the non-inclusion of other indicators such as pathological types.

RECOvER, reporting on ERAS compliance, outcomes, and elements research; IRB, Institutional Review Board; PPOI, prolonged postoperative ileus; ICU, intensive care unit.

### 2.3 Statistical method

SPSS22.0 statistical software was used for data processing and analysis, the measurement data was expressed in $\left(\bar{x}\pm s\right)$, and *t*-test of independent samples was used for comparison between the two groups. Count data were expressed as a percentage, and *X*^2^ test was used. *P* < 0.05 indicates a statistically significant difference.

## 3 Results

### 3.1 Demographic and surgical characteristics of patients in both groups

From December 2019 to December 2022, the data of 594 patients were continuously entered into the EIAS database, and the selection process is as shown in Figure 1. Of the 498 patients who met the inclusion criteria, 255 underwent RC surgery and 243 underwent LC surgery. The detailed demographic characteristics of these patients are shown in Table 3. In terms of demographic variables, there was no significant difference between the two groups (*P* > 0.05).

**Table 3:** Demographic characteristics of patients.

	Right colectomy (*n* = 255)	Left colectomy (*n* = 243)	*P*
Age (years)	60.2 ± 13.5	61.5 ± 14.2	0.536
Gender (male, *N*)	135	114	0.325
BMI (kg/m^2^)	22.7 ± 2.8	22.5 ± 2.7	0.892
ASA * classes I–II (*N*)	216	195	0.103
Cardiac disease (*N*)	9	6	0.189
Pulmonary disease (*N*)	21	9	0.253
Diabetes (*N*)	48	30	0.447
Smoking (*N*)	54	51	0.572
Mechanical bowel preparation (*N*)	15	9	0.634
Previous abdominal surgery (*N*)	111	93	0.670
Preoperative Education (*N*)	234	222	0.509
Pathological TNM staging (*N*)			0.368
I	27	21	
II	117	105	
III	111	117	
Haemoglobin (g/L)	112.2 ± 8.7	113.1 ± 8.5	0.602
Albumin (g/L)	33.7 ± 4.2	34.4 ± 3.9	0.590

ASA, American Society of Anesthesiologists.

In terms of intraoperative variables, fluid doses were higher in the LC group than in the RC group (*P* < 0.05), and there was no significant difference between them in terms of operative time, blood loss, need for open surgery, peritoneal contamination, epidural catheter placement, or opioid use (*P* > 0.05, Table 4).

**Table 4:** Intraoperative results.

	Right colectomy (*n* = 255)	Left colectomy (*n* = 243)	*P*
Epidural anesthesia (*N*)	237	204	0.320
Total fluids (ml)	1,452 ± 327	1,509 ± 364	0.092
Operative time (min)	159 ± 43	167 ± 51	0.079
Blood loss (ml)	215 ± 31	224 ± 45	0.125
Conversion to open surgery (*N*)	9	6	0.189
Peritoneal soiling (*N*)	12	9	0.365
Opioids given (*N*)	174	168	0.419

### 3.2 Postoperative functional recovery in both groups

Compared with the RC group, the LC group had a higher intake of oral liquid at the second POD, and faster first flatulence (*P* < 0.05). 30 (11.76%) RC patients required nasogastric tube insertion, while only 3 (1.23%) in the LC group required the same (*P* < 0.05, Table 5). PPOIs occurred in 48 (18.82%) and 29 (11.93%) patients in the RC and LC groups, respectively (*P* < 0.05).

**Table 5:** Postoperative functional recovery.

	Right colectomy (*n* = 255)	Left colectomy (*n* = 243)	*P*
Oral fluid intake 1st POD (ml)	1,945 ± 523	2,042 ± 610	0.102
Oral fluid intake 2nd POD (ml)	1,461 ± 415	1,821 ± 367	0.003
Oral energy intake 1st POD (kcal)	552 ± 128	586 ± 135	0.153
Nausea VAS 1st POD (*N*)	24	18	0.215
Nausea VAS 2nd POD (*N*)	36	18	0.174
Nausea VAS 3rd POD (*N*)	21	12	0.129
Nasogastric tube (*N*)	30	3	0.011
PPOI	48	29	0.010
Opioid use first 48 h (*N*)	33	21	0.079
First flatus passage (days)	2.3 ± 0.4	1.5 ± 0.3	0.041
First stool passage (days)	2.8 ± 0.5	2.0 ± 0.4	0.059
Tolerate solid food (days)	2.1 ± 0.3	2.2 ± 0.4	0.190
Median post-operative stay (days)	5.5 ± 0.8	5.4 ± 0.7	0.188

POD, postoperative day.

### 3.3 Postoperative complication in both groups

**Figure 2:**
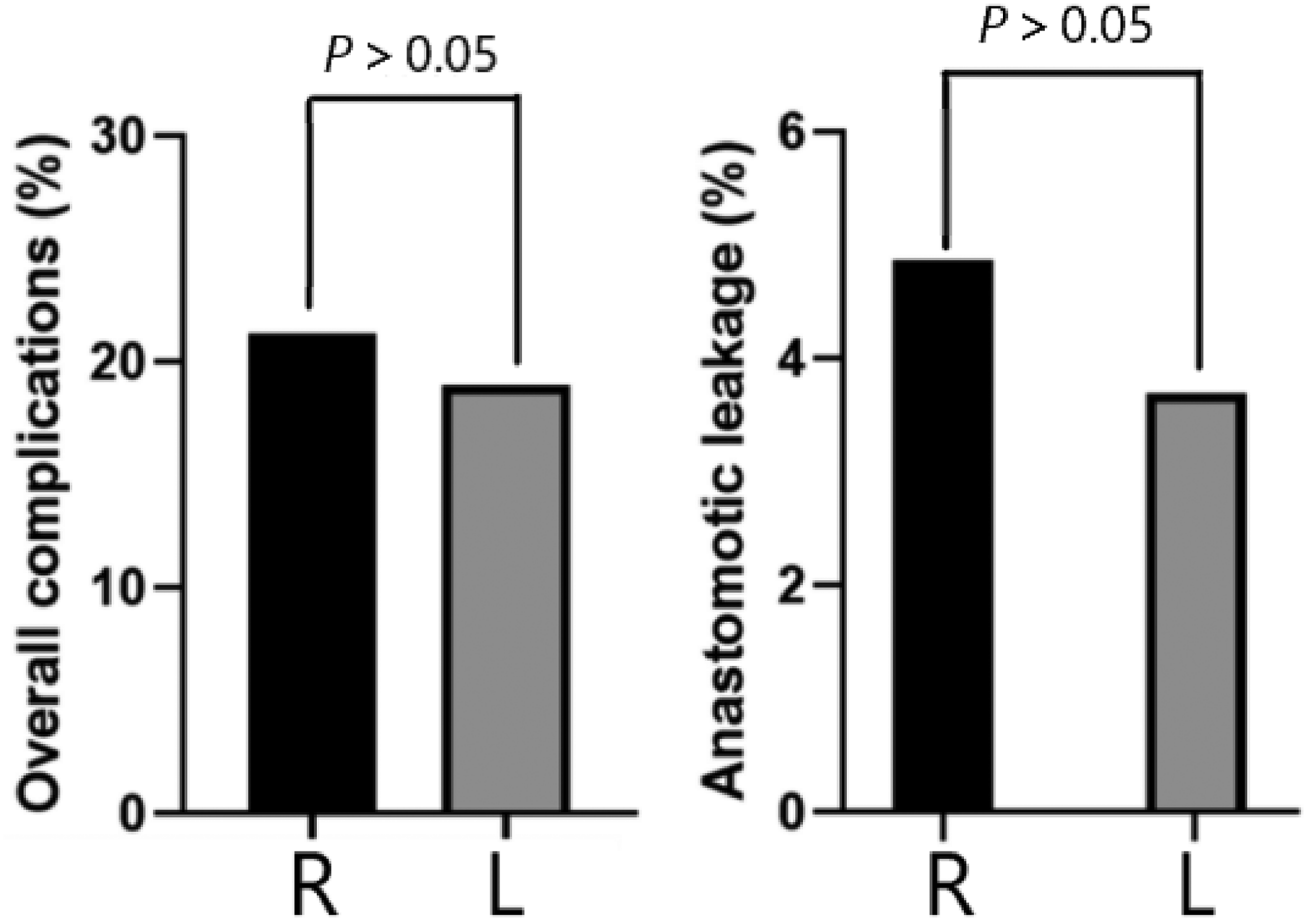
Postoperative complication.

In terms of postoperative morbidity, 52 (20.39%) and 45 (18.52%) patients in the RC and LC groups developed postoperative complications (*P* = 0.150), respectively. In the RC group, 34 (13.33%) had minor complications (6 cases of urinary tract infection, 9 cases of superficial infection at surgical site, 1 cases of acute renal failure, 18 cases of hospital-acquired pneumonia) and 18 (7.06%) had serious complications: including 12 cases (4.71%) of anastomotic leakage that required follow-up surgical intervention, 3 cases of percutaneous drainage of intra-abdominal abscess, and 3 cases of deep wound dehiscence that required surgical revision. In the LC group, 30 (12.35%) patients had minor complications (4 cases of urinary retention, 3 cases of urinary tract infections, and 8 cases of superficial surgical site infections, 15 cases of hospital-acquired pneumonia), 15 (6.17%) patients had serious complications: including 9 cases of (3.70%) anastomotic leakage that required surgical reintervention, and 6 cases of percutaneous drainage of intra-abdominal abscesses. See Figure 2 for details.

## 4 Discussion

Over the past two decades, there have been two major improvements/fundamental developments in the perioperative care of patients having colon surgeries, namely the introduction of laparoscopic surgery [formerly known as Fast Track (FT) surgery] and the implementation of ERAS protocols. Compared to open surgery, laparoscopic surgery results in a significantly shorter median post-operative stay and much less postoperative morbidity and pain. As reported in multiple randomized controlled trials, this is due to less surgical stress response (14, 15). And it is also found in several randomized studies evaluating the effect of ERAS protocols on the outcomes of elective colorectal surgery that the implementation of ERAS helps reduce complications and median post-operative stay and accelerate postoperative recovery (16, 17).

Studies have shown that improvement in postoperative recovery depends primarily on intraoperative factors (e.g., minimally invasive surgery and afferent nerve block) and postoperative interventions (e.g., early nutritional preparation and mobilization after “fast-track” surgery) (18). The purpose of postoperative interventions is to promote the recovery of physical functions and accelerate recovery. However, studies have shown that due to poor health and psychological burden (fear of interfering with the healing process and anxiety about the possible additional treatment of the underlying disease) in the postoperative period, it may not be the best time to ask patients to make significant changes in nutritional intake and exercise (19). In comparison, interventions in the preoperative phase (both physically and psychologically) may be more helpful for postoperative recovery and relieving some of the emotional distress associated with the anticipation and recovery process of surgery. Some studies have also shown that regardless of motor skills, patients who improved their physical functions through exercise before surgery recovered better thereafter (20). However, one-third of patients experienced deterioration before surgery. Despite exercises, these patients were still at risk of prolonged postoperative recovery. Poor preoperative physical conditions (fatigue, malnutrition, and physical performance), anxiety and depression are also important confounding factors that can lead to prolonged recovery (21, 22). These results indicate that exercise training alone is not enough to reduce stress responses in all patients, and it is also necessary to consider factors that can promote adaptation to exercises, such as nutritional preparation and coping behaviors. Based on the foregoing and our experience, we believe that personalized postoperative rehabilitation plans should be developed for different patients and rehabilitation treatment should be carried out according to the specific conditions of the patients. Early walking exercise, diet recovery, and aggressive pain control are important components of ERAS, and they have been shown to aid recovery after colorectal cancer surgery. Therefore, the ERAS protocols covered in this study all encourage patients to do walking exercise, active diet recovery, and pain control as early as possible.

In our study, there were no significant differences between the groups in terms of time to first ventilation or bowel movement and time to tolerate solid foods, and the composite marker PPOI showed a clear trend towards slow bowel recovery after RC. Postoperative ileus (POI) has no internationally accepted clinical definition. As a quasi-physiological state of coordinated bowel motility arrest after surgical intervention, it usually disappears on the third day after laparoscopic surgery (23). PPOI refers to the inadequate recovery of POI on day 3 postoperatively, which impedes efficient delivery of intestinal contents or tolerance of oral intake of food. We consider this metric to be more reliable than some other commonly used indicators: the timing of first ventilation and stool is an inaccurate marker of bowel recovery, as patients may pass a small amount of gas or stool on the first day postoperatively but still have paralytic ileus for two to three days after surgery (24). Therefore, understanding the potential dangers of PPOIs is clinically relevant and can help surgeons avoid PPOIs through specific measures such as the use of prokinetic drugs and adjustment of postoperative food intake. In addition, the use of MBP may cause a delay in the first bowel movement. Times to solid food tolerance and appetite recovery may also be unreliable and subjective markers of bowel function recovery, as they lack quantitative definitions (25). Therefore, if these markers were used alone, the sensitivity to measure intestinal recovery would be low, and the curve of differences between groups would flatten. For these reasons, we believe that composite PPOIs are more adequate in terms of the condition assessment of patients. In terms of intraoperative variables, fluid doses were higher in the LC group than in the RC group (*P* < 0.05). Compared with the RC group, the LC group had a higher intake of oral liquid at the second POD, and faster first flatulence (*P* < 0.05). Postoperative complications occurred in 28.24% and 18.52% of patients with RC and LC, respectively, with no significant difference (*P* > 0.05). The main purpose of perioperative bowel preparation for colon cancer surgery is to rid the large bowel of solid feces and lower the bacterial content. However, this practice in fact liquefies the feces which increases the risk of surgical spilling and does not reduce the number of bacterial organisms in the bowel. This has been phased out in our latest ERAS agreement. The purpose of placing abdominal drainage is to observe and assist in the treatment of abdominal bleeding, anastomotic leakage, chyle leak, etc. after the surgery (to ensure adequate drainage and increase the success rate of conservative treatment). Therefore, in this study, there was no difference in the overall incidence of complications between the two groups, but there were differences between them in some specific complications, such as those requiring nasogastric tube insertion and the proportion of PPOI.

### 4.1 Study limitations

First, as a small-sample, single-center retrospective study, this research covers only a small number of cases, and the conclusion needs to be validated by further multi-cohort studies. Second, in terms of observation indicators, this study lacks comprehensiveness due to the non-inclusion of other indicators such as pathological types.

In summary, despite the wide application and proven effects of ERAS protocol in the field of CRC surgery, we should develop more individualized ERAS protocols by also considering different tumor locations and surgical protocols.

## Data Availability

The raw data supporting the conclusions of this article will be made available by the authors, without undue reservation.

## References

[1] Siegel RL Giaquinto AN Jemal A. Cancer statistics. CA Cancer J Clin. (2024) 74(1):12–49. 10.3322/caac.21820 38230766

[2] Geltzeiler CB Rotramel A Wilson C Deng L Whiteford MH Frankhouse J. Prospective study of colorectal enhanced recovery after surgery in a community hospital. JAMA Surg. (2014), 149(9):955–61. 10.1001/jamasurg.2014.675 25054315

[3] Bourgouin S Monchal T Schlienger G Franck L Lacroix G Balandraud P. Eligibility criteria for ambulatory colectomy. J Visc Surg. (2022) 159(1):21–30. 10.1016/j.jviscsurg.2020.11.012 33349570

[4] van Rooijen S Carli F Dalton S Thomas G Bojesen R Le Guen M Multimodal prehabilitation in colorectal cancer patients to improve functional capacity and reduce postoperative complications: the first international randomized controlled trial for multimodal prehabilitation. BMC Cancer. (2019) 19(1):98. 10.1186/s12885-018-5232-6 30670009 PMC6341758

[5] Bakker N Cakir H Doodeman HJ Houdijk AP. Eight years of experience with enhanced recovery after surgery in patients with colon cancer: impact of measures to improve adherence. Surgery. (2015) 157(6):1130–6. 10.1016/j.surg.2015.01.016 25791027

[6] Martínez AB Longás J Ramírez JM. A model for lymphocyte activation in open versus laparoscopic surgery in colorectal cancer patients in enhanced recovery after surgery (ERAS) protocols. Int J Colorectal Dis. (2017) 32(6):913–6. 10.1007/s00384-016-2731-2 27900463

[7] Lohsiriwat V Lertbannaphong S Polakla B Riansuwan W. Implementation of enhanced recovery after surgery and its increasing compliance improved 5-year overall survival in resectable stage III colorectal cancer. Updates Surg. (2021) 73(6):2169–79. 10.1007/s13304-021-01004-8 33599947

[8] Jones D Musselman R Pearsall E McKenzie M Huang H McLeod RS. Ready to go home? Patients’ experiences of the discharge process in an enhanced recovery after surgery (ERAS) program for colorectal surgery. J Gastrointest Surg. (2017) 21(11):1865–78. 10.1007/s11605-017-3573-0 28932946

[9] De Crignis L Slim K Cotte E Meillat H Dupré A. Impact of surgical indication on patient outcomes and compliance with enhanced recovery program for colorectal surgery: a francophone multicenter retrospective analysis. J Surg Oncol. (2020) 122(5):928–33. 10.1002/jso.26097 32627198

[10] Kim MK Kim JG Lee G Won DD Lee YS Kye BH Comparison of the effects of an ERAS program and a single-port laparoscopic surgery on postoperative outcomes of colon cancer patients. Sci Rep. (2019) 9(1):11998. 10.1038/s41598-019-48526-1 31427651 PMC6700146

[11] Lohsiriwat V Jitmungngan R Chadbunchachai W Ungprasert P. Enhanced recovery after surgery in emergency resection for obstructive colorectal cancer: a systematic review and meta-analysis. Int J Colorectal Dis. (2020) 35(8):1453–61. 10.1007/s00384-020-03652-5 32572602

[12] Pedziwiatr M Mavrikis J Witowski J Adamos A Major P Nowakowski M Current status of enhanced recovery after surgery (ERAS) protocol in gastrointestinal surgery. Med Oncol. (2018) 35(6):95. 10.1007/s12032-018-1153-0 29744679 PMC5943369

[13] Meillat H Brun C Zemmour C de Chaisemartin C Turrini O Faucher M Laparoscopy is not enough: full ERAS compliance is the key to improvement of short-term outcomes after colectomy for cancer. Surg Endosc. (2020) 34(5):2067–75. 10.1007/s00464-019-06987-5 31385073

[14] Gustafsson UO Scott MJ Hubner M Nygren J Demartines N Francis N Guidelines for perioperative care in elective colorectal surgery: enhanced recovery after surgery (ERAS®) society recommendations: 2018. World J Surg. (2019) 43(3):659–95. 10.1007/s00268-018-4844-y 30426190

[15] Pellegrino L Pagano E Allaix ME Morino M Muratore A Massucco P Perioperative care in colorectal cancer surgery before a structured implementation program of the ERAS protocol in a regional network. The piemonte EASY-NET project. Healthcare (Basel). (2021) 10(1):72. 10.3390/healthcare10010072 35052236 PMC8775376

[16] Chen JS Sun SD Wang ZS Cai TH Huang LK Sun WX The factors related to failure of enhanced recovery after surgery (ERAS) in colon cancer surgery. Langenbecks Arch Surg. (2020) 405(7):1025–30. 10.1007/s00423-020-01975-z 32870334

[17] Tejedor P González Ayora S Ortega López M León Arellano M Guadalajara H García-Olmo D Implementation barriers for enhanced recovery after surgery (ERAS) in rectal cancer surgery: a comparative analysis of compliance with colon cancer surgeries. Updates Surg. (2021) 73(6):2161–8. 10.1007/s13304-021-01115-2 34143398

[18] Salem ME Yin J Goldberg RM Pederson LD Wolmark N Alberts SR Evaluation of the change of outcomes over a 10-year period in patients with stage III colon cancer: pooled analysis of 6,501 patients treated with fluorouracil, leucovorin, and oxaliplatin in the ACCENT database. Ann Oncol. (2020) 31(4):480–6. 10.1016/j.annonc.2019.12.007 32085892 PMC10688027

[19] Ripollés-Melchor J Abad-Motos A Zorrilla-Vaca A. Enhanced recovery after surgery (ERAS) in surgical oncology. Curr Oncol Rep. (2022) 24(9):1177–87. 10.1007/s11912-022-01282-4 35403970

[20] Meillat H Braticevic C Zemmour C Brun C Cécile M Faucher M Real-world implementation of a geriatric-specific ERAS protocol in patients undergoing colonic cancer surgery. Eur J Surg Oncol. (2021) 47(5):1012–8. 10.1016/j.ejso.2020.11.128 33261952

[21] Bellato V An Y Cerbo D Campanelli M Franceschilli M Khanna K Feasibility and outcomes of ERAS protocol in elective cT4 colorectal cancer patients: results from a single-center retrospective cohort study. World J Surg Oncol. (2021) 19(1):196. 10.1186/s12957-021-02282-7 34215273 PMC8253238

[22] Skelton WP 4th Franke AJ Iqbal A George TJ. Comprehensive literature review of randomized clinical trials examining novel treatment advances in patients with colon cancer. J Gastrointest Oncol. (2020) 11(4):790–802. 10.21037/jgo-20-184 32953161 PMC7475336

[23] Kloosterman WP Coebergh van den Braak RRJ Pieterse M van Roosmalen MJ Sieuwerts AM Stangl C A systematic analysis of oncogenic gene fusions in primary colon cancer. Cancer Res. (2017) 77(14):3814–22. 10.1158/0008-5472.CAN-16-3563 28512242

[24] Vendler MMI Haidari TA Waage JE Kleif J Kristensen B Gögenur I Incidence of venous thromboembolic events in enhanced recovery after surgery for colon cancer: a retrospective, population-based cohort study. Colorectal Dis. (2017) 19(11):O393–401. 10.1111/codi.13910 28980383

[25] Sánchez-Iglesias JL Gómez-Hidalgo NR Bebia V Ramirez JM Pérez-Benavente A Nelson G Discontinuation of mechanical bowel preparation in advanced ovarian cancer surgery: an enhanced recovery after surgery (ERAS) initiative. Clin Transl Oncol. (2023) 25(1):236–42. 10.1007/s12094-022-02934-4 36273061

